# Bibliometric analysis of global research trends on post-stroke pneumonia: Current development status and research Frontiers

**DOI:** 10.3389/fpubh.2022.950859

**Published:** 2022-08-02

**Authors:** Xiangfei Li, Jiahui Yu, Chang Shu

**Affiliations:** ^1^School of Economics and Management, Tiangong University, Tianjin, China; ^2^Tianjin Key Laboratory of Cerebral Vascular and Neurodegenerative Diseases, Tianjin Neurosurgical Institute, Tianjin Huanhu Hospital, Tianjin, China

**Keywords:** post-stroke pneumonia, bibliometric analysis, CiteSpace, VOS viewer, hotspots

## Abstract

**Background:**

As one of the most common complications of stroke, post-stroke pneumonia significantly increases the incidence of adverse outcomes, mortality, and healthcare costs for patients with stroke. As the field of post-stroke pneumonia has gained interest in the recent years, there has been an increasing number of publications on post-stroke pneumonia research worldwide. Therefore, a more comprehensive understanding of the field is needed now. This paper is intended to analyze the research status and detect the research frontiers in this field.

**Methods:**

VOS viewer, CiteSpace, and the online scientometric platform (https://bibliometric.com/) were the main visualization tools used in this paper. They were used to perform citation analysis of countries/institutions, co-citation analysis of authors/journals/references, co-authorship analysis of authors, co-occurrence analysis of keywords, and citation bursts analysis of references.

**Results:**

The number of publications in this field has increased rapidly since 2010 and is expected to continue to increase in the next few years. The countries contributing most to post-stroke pneumonia research were the USA, China, and Germany. The most productive institution was Harvard University, followed by Humboldt University of Berlin, Charité Universitätsmedizin Berlin, and Free University of Berlin from Germany. Meanwhile, the German authors Meisel A, Meisel C, and Dirnagl U, who have contributed significantly to this field, were all associated with these three German institutions. The high-quality and high output journal was *STROKE*. In the coming years, the hot topic keywords “risk & risk-factors,” “outcome & impact,” “management & guidelines,” and “predictors” will gain more attention in this field. Finally, hot keywords were grouped into four clusters in this paper: cluster 1 (risk-factors studies of post-stroke pneumonia), cluster 2 (clinically relevant studies of post-stroke pneumonia), cluster 3 (mechanism studies of post-stroke pneumonia), and cluster 4 (care studies of post-stroke pneumonia).

**Conclusion:**

This study shows the knowledge structure and evolution of the field of post-stroke pneumonia research and predicts research trends through visualization analysis. The future trend of post-stroke pneumonia research will gradually shift from clinical and mechanistic studies to treatment and prevention studies.

## Introduction

Post-stroke pneumonia is one of the most common complications of stroke, and it is estimated that about one in ten patients with stroke will develop pneumonia (1). The term “stroke-associated pneumonia” (SAP) was first introduced by Hilker et al. at the University Hospital Cologne, Germany, who found that approximately 75% of all post-stroke pneumonia occurred within the first 72 h of hospitalization (2). In 2015, the pneumonia in stroke consensus (PISCES) study group, a multidisciplinary group of experts, published its first consensus to clarify the definition and diagnostic criteria for SAP, defining SAP as a lower respiratory tract infection occurring during the week of stroke and recommending the modified Centers for Disease Control and Prevention (CDC) criteria as the diagnostic criteria for SAP (3), which are now well accepted internationally for the diagnosis of SAP (4, 5). In addition, the presence of post-stroke pneumonia significantly increases mortality in patients. A large community-based study of stroke outcomes in 14,293 participants found that patients who develop pneumonia after stroke have three times the mortality rate of those who do not have pneumonia (6). Other studies have shown that pneumonia is associated with one-third of early deaths and one-fifth of adverse stroke outcomes (7, 8).

The occurrence of pneumonia after stroke also significantly increases the cost of treatment. It has been investigated that the annual cost of patients with stroke-associated pneumonia during hospitalization in the USA was nearly $459 million (9); in a related study in the UK, the occurrence of stroke-associated pneumonia resulted in an adjusted incremental additional cost of £5,817 per patient (10); in Argentina, patients with intracerebral hemorrhage (ICH) who developed pneumonia during hospitalization spent $16,893 more than those who did not, and for patients with ischemic stroke (IS), those who developed pneumonia spent $36,149 more (11).

The number of publications in the field of post-stroke pneumonia has increased in the recent years. In the face of this vast amount of the literature, it is important to explore the history of the field, to understand the main research efforts, and to identify where the hotspots of research have been over the years and where the future trends lie. Bibliometric analysis is a popular quantitative analysis method in many disciplines, which has been adopted by many scholars in the field of health so far. For example, it has been used in renal fibrosis in diabetic kidney disease (12), personal protective equipment in COVID-19 (13), and macrophages associated with acute lung injury (14). By visualizing the content and citations of published articles, this type of article can help readers gain a better understanding of research hotspots and identify future research directions in a particular research discipline. It can be said that they have offered significant reference value for subsequent research in a discipline (15–17). However, as far as we know, there is no complete bibliometric study in the field of post-stroke pneumonia. Bibliometrics offers a visual path that can be used to understand the structure and evolution trend of a research field in general (18, 19), which is important for us to grasp the knowledge framework and research hotspots in the field of post-stroke pneumonia on a global scale.

This study was based on a bibliometric analysis of the publications about post-stroke pneumonia from 1998 to 2021 using the Web of Science database with the software of CiteSpace and VOS viewer. Specifically, the goal of this paper was to give an outline of the current state of research on post-stroke pneumonia and the main contributors (including analysis of annual publications, countries, institutions, journals, authors, etc.), to summarize the main research hotspots and look forward to the field's future. This article offers a fresh approach to learning this field from a professional standpoint and allows the reader to gain a better grasp of the present state of research and its research trends.

## Methods

### Data source

In this paper, Science Citation Index Expanded (SCI-EXPANDED) and Social Science Citation Index (SSCI) from the Web of Science Core Collection (WoSCC), the most influential, authoritative, and comprehensive database in the world, were selected as the data sources. During the research process, we also searched the database of PubMed, resulting in a total of 200 documents. After comparing the two databases (PubMed and WoSCC), it was found that 180 articles overlapped, and the remaining 20 articles had low relevance to the topic. After a comprehensive comparison, we finally chose the WoSCC as the final database source.

### Data collection

We conducted a literature search and data download on 16 June 2022, to reduce bias due to frequent database updates. Details of the search strategy are shown below: The search term was “TS = (post-stroke pneumonia OR poststroke pneumonia OR stroke-associated pneumonia OR pneumonia after stroke).” The timespan was from 01-01-1998 to 31-12-2021, the type of literature was restricted to “review” and “article,” and the language was restricted to English.

### Data extraction

**Figure 1:**
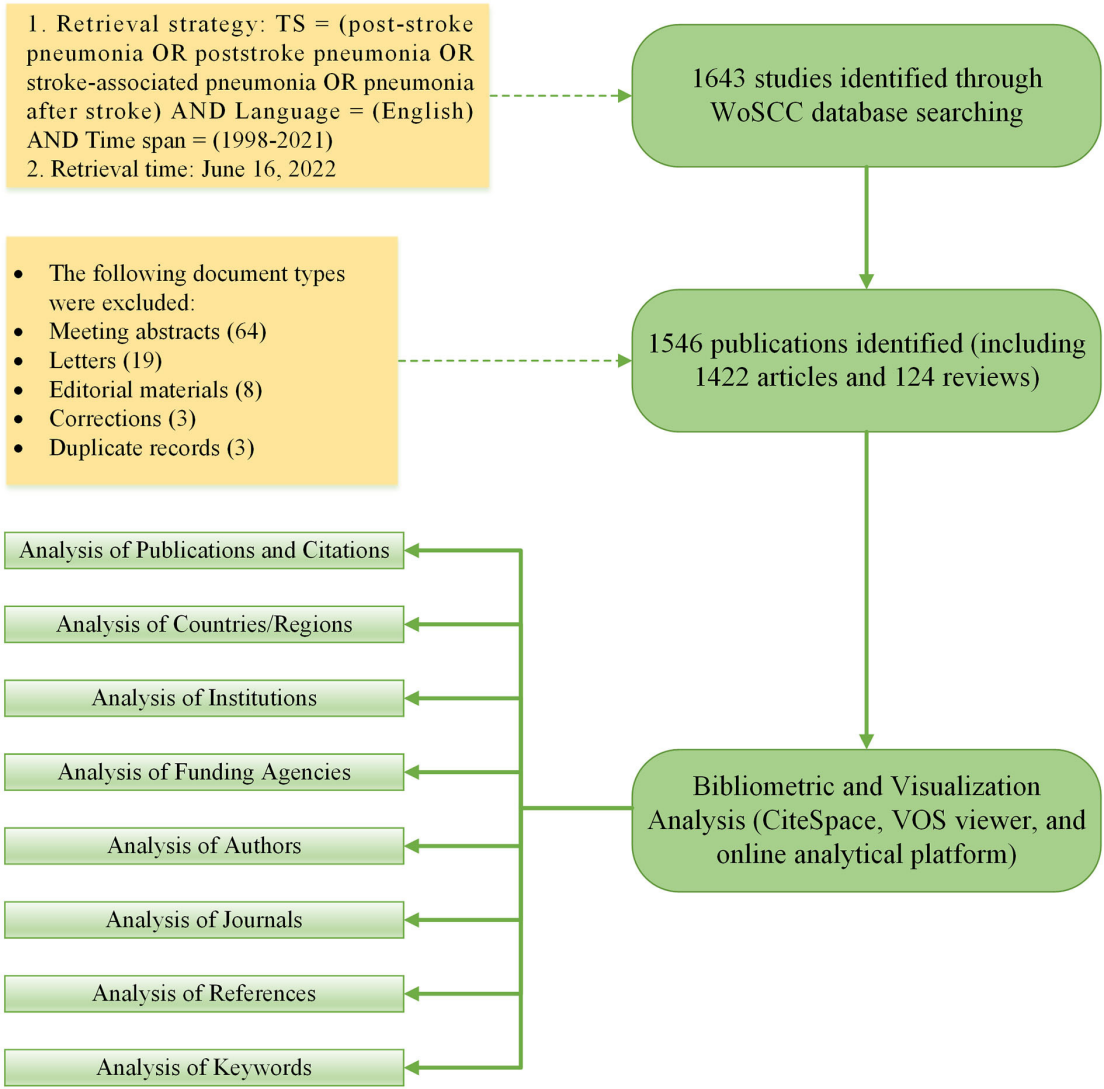
Flowchart for the identification and selection of publications.

A total of 1,549 documents were retrieved from the WoSCC database according to the above retrieval strategy. A number of three duplicate records were found after manual checking, and then, we removed them to ensure that the results were as scientific as possible. The remaining 1,546 documents (including 1,422 original research articles and 124 reviews) were exported, checked “Full Record and Cited References,” and saved as plain text files and tab delimited files. The “Full Record and Cited References” options, including titles, authors, abstracts, and cited references, can provide the most comprehensive information on the literature for more detailed analysis at a later stage of the study. Figure 1 depicts the specific literature screening procedure.

### Data analysis

#### Web of Science self-contained analysis function

The analysis function provided by the Web of Science database was used to comprehensively mine and analyze the retrieved literature in terms of country/region, institution, author, etc., and to conduct a statistical analysis of the data using EXCEL according to the purpose and content of the study.

#### Analysis with VOS viewer

VOS viewer is a computer program for building and viewing bibliometric maps (20) and is used in different fields because of its excellent view effect (18, 19, 21, 22). The version used in this paper is VOS viewer 1.6.17. In the VOS viewer software, every node represents a different parameter, such as a country/region, journal, author, keyword, and so on. The weight of a parameter, such as the number of published articles, the number of citations, or the frequency of occurrence, determines the size of a node, and the higher the weight, the larger the node. The cluster to which the nodes and lines belong determines their color, with nodes of the same color acting as a cluster and different clusters being distinguished by different colors. The distance between nodes indicates the closeness and similarity between subject terms, and the lines between nodes indicate links. The strength of links is assessed quantitatively through the total link strength (TLS), which is the sum of all the link strengths. It can reflect the strength of co-authorship and co-citation linkages between authors, references, or institutions (23, 24).

#### Analysis with CiteSpace

CiteSpace is a JAVA application for visualizing information (25). CiteSpace V 5.8.R3 was used to visualize the progress of research in a particular field using information such as authors, titles, keywords, abstracts, citations, and so on. The results of statistical filtering by different years and data collation calculations in Excel were used to analyze the trends in the field of post-stroke pneumonia research from 1998 to 2021. In the network results, each node represents a keyword, an author, a country, etc. The size of the node indicates the frequency of occurrence of the node, and the thickness of the line between nodes indicates the strength of co-occurrence. Betweenness centrality (BC) and burst detection are two important concepts involved in this study. The betweenness centrality (BC) is an important indicator for determining the importance of nodes in the network (25). The higher the BC value of a node, the greater the influence of the node. The burst detection of references reflects the specific duration of articles in which the burst occurred (26). Higher strength values indicate that the node has a stronger frequency burst in a certain period. In other words, this type of theme research is more popular in this period.

#### Bibliometric analysis with an online platform

In addition to the methodologies described above, a website (https://bibliometric.com/) was supplemented for the analysis of international cooperation between countries.

#### Procedures for analysis

In this study, citation analysis, co-citation analysis, co-authorship, and co-occurrence analysis were mainly used to visualize the countries/regions, authors, references, keywords, etc. Citation analysis is able to express the relationship between cited items through the number of citations, and there is a link between the number of citations of a study and the intrinsic value of the study (27). It is used in this paper to identify key countries/regions and institutions. Co-citation analysis was proposed by Small (28) to detect the intellectual structure of a research topic. Co-citation occurs when two articles are cited by a third article at the same time (22). In comparison with citation, co-citation is a more accurate mapping approach (29). It is used in this study for references, journals, and authors. Co-authorship analysis refers to the number of co-authored documents to reflect the collaboration among authors and the contribution and influence of core authors (30). Co-occurrence of keywords means the number of times they appear together, which can help the one to understand the distribution of high-frequency keywords (20).

During the analysis, the basic parameters of the software VOS viewer are as follows: visualization weights (citations), normalization method (association strength), clustering resolution (1.00), and minimum cluster size (1). To use CiteSpace to mine literature information, we first set the parameters: time slicing (1998–2021), years per slice (1 year), and selection criteria (g-index, k = 25). In the “Node Types” module, we selected the items to be analyzed, set the rest of the parameters (Links and Pruning) to the default settings, and then ran the software to calculate the centrality.

## Result

### Analysis of publications and citations

**Figure 2:**
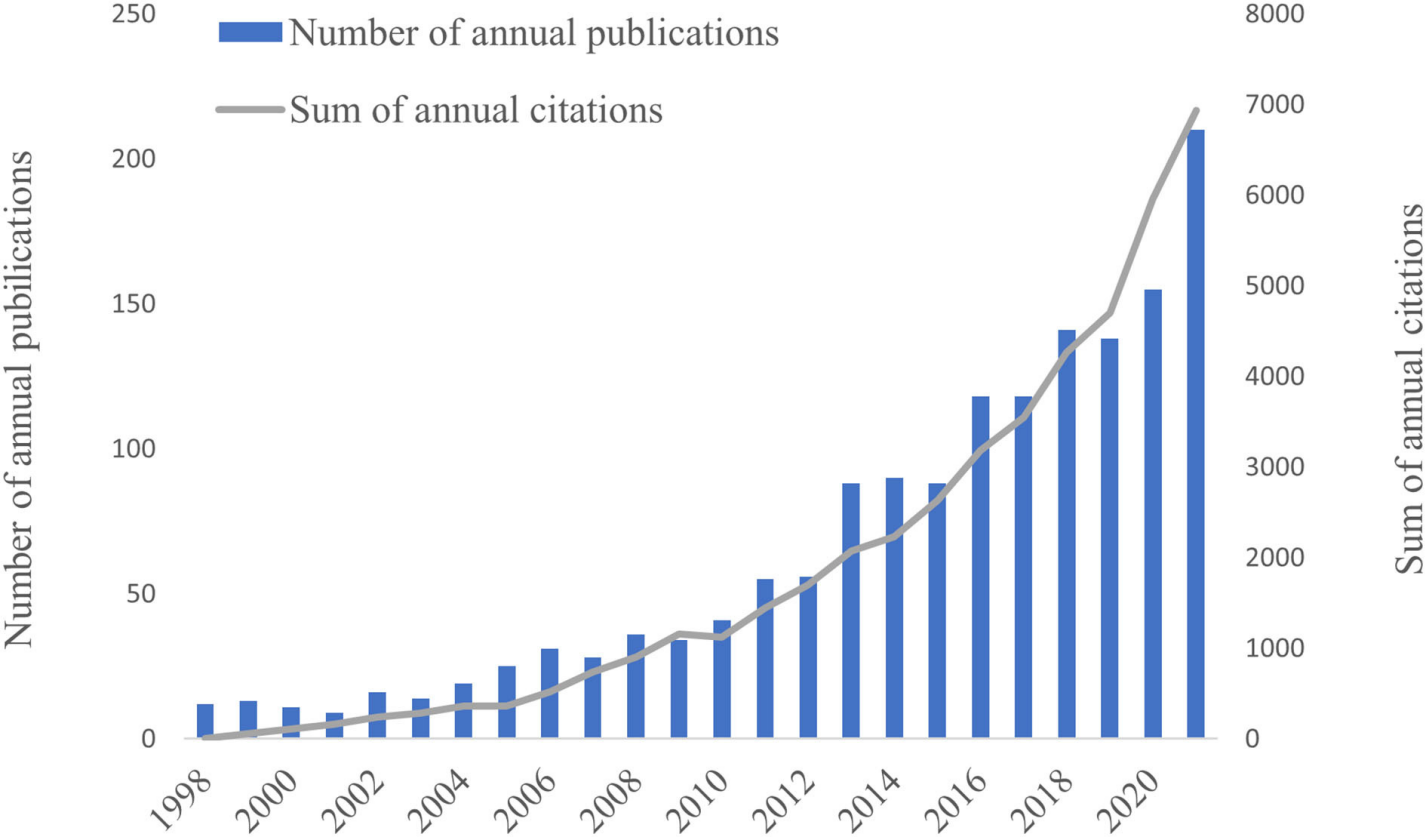
The number of annual publications and the sum of annual citations on post-stroke pneumonia research from 1998 to 2021.

According to Figure 2, the number of annual publications and citations in the WoSCC database generally showed an increasing trend between 1998 and 2021. Prior to 2010, research on post-stroke pneumonia was relatively slow to develop. None of them had more than 40 annual publications and 1,200 annual citations. After 2010, the number of annual publications and citations began to increase gradually, with a stepwise rise in the number of publications and continuous growth in the number of citations. In 2021, the number of annual articles and citations reached a peak of 210 articles and 6,929 citations. As can be seen, there is a growing number of relevant studies in this field, and gradually more attention is being paid to it.

### Analysis of countries/regions

A total of 75 countries contributed to publications on post-stroke pneumonia in the WoSCC database. The top 10 countries/regions contributing to publications are shown in Table 1. Approximately 55.56% of publications in the field of post-stroke pneumonia were from the top three countries with the most publications: the USA, China, and Germany. Of these, the USA (31.50%) was the country with the most publications, followed by China (15.20%) and Germany (11.77%). The United States was still in first place in terms of total citations and H-index, whereas the country with the highest average citations was Canada. It should be noted that due to collaboration between authors, some articles would belong to different countries at the same time in the statistical attribution.

**Table 1:** The top 10 countries/regions contributing to publications about post-stroke pneumonia.

**Rank**	**Countries /Regions**	**Article counts**	**Percentage (*n*/1,546)**	**Total citations**	**Average citations per article**	**H-index**	**TLS**
1	USA	487	31.50%	18,941	38.89	73	2,852
2	China	235	15.20%	3,369	14.34	25	2,275
3	Germany	182	11.77%	9,688	53.23	47	3,325
4	England	132	8.54%	5,436	41.18	41	2,264
5	Japan	112	7.24%	2,404	21.46	24	536
6	Taiwan, China	93	6.02%	1,401	15.06	21	456
7	Canada	76	4.92%	6,739	88.67	30	1,037
8	Australia	68	4.40%	2,289	33.66	25	939
9	South Korea	64	4.14%	1,040	16.25	15	465
10	Italy	59	3.82%	2,700	45.76	24	936

**Figure 3:**
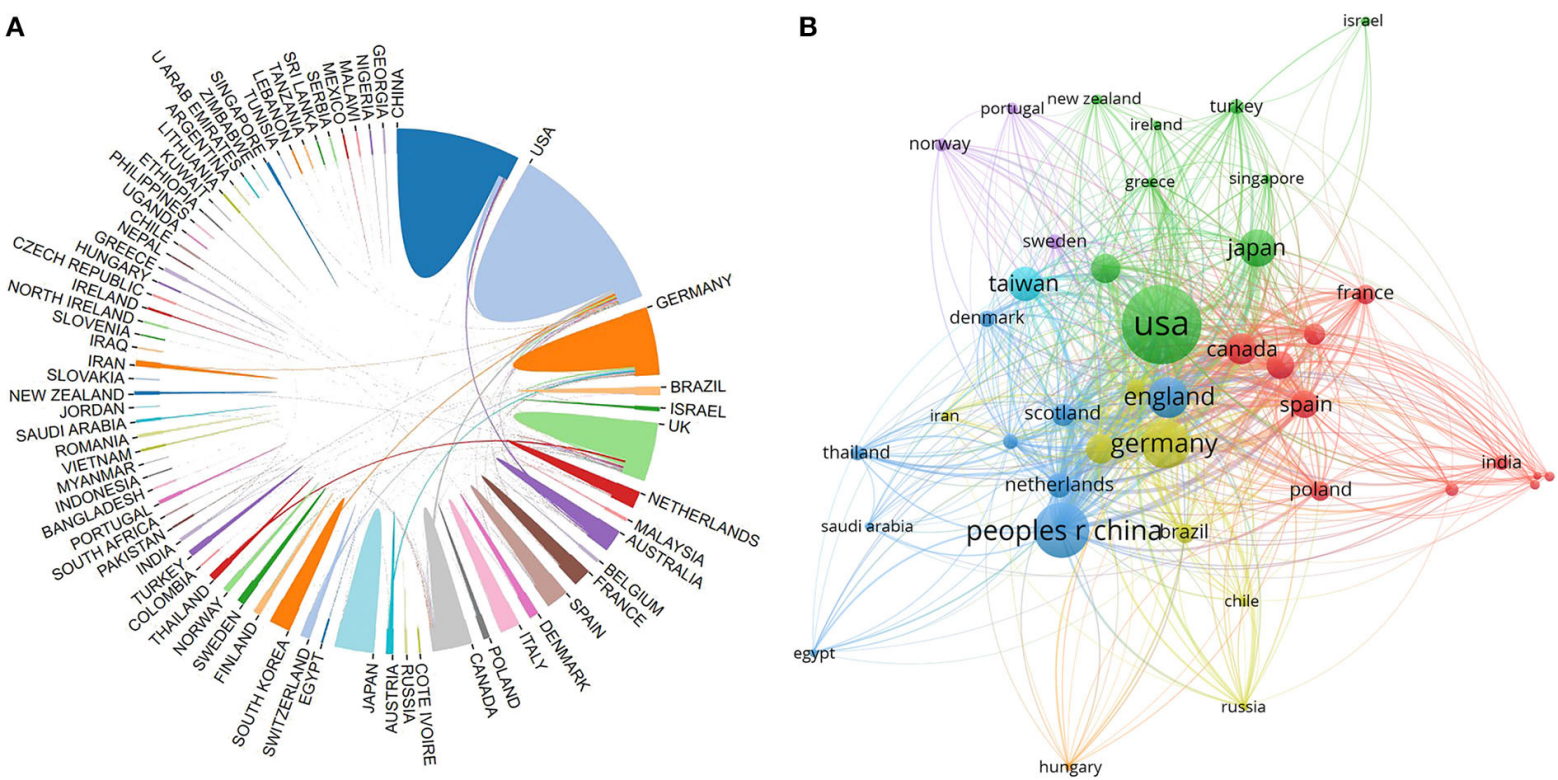
**(A)** A map of international collaboration among countries/regions. **(B)** The country's citation network visualization map generated by VOS viewer.

International collaboration among countries/regions was analyzed in Figure 3A, where a thicker line between two countries indicates stronger cooperation. As the figure shows, countries such as the United States, the Unitded Kingdom, and Germany were more connected to other countries. Figure 3B shows the country's citation network visualization map. By adjusting the parameters of the graph, a total of 41 countries and regions with at least five documents were selected. The countries with a total link strength (TLS) of over 2,000 were Germany (TLS = 3,325), the United States (TLS = 2,852), and China (TLS = 2,275). This indicates that these three countries have a broader range of influence in the international arena. In general, the United States, Germany, and England were among the top international contributors to post-stroke pneumonia research, with more publications and higher-quality papers. China and Japan each possessed a substantial number of publications, but the quality of publications still needs to be improved, and scientific research in this area should be strengthened even further.

### Analysis of institutions

A total of 2,300 institutions had published articles on post-stroke pneumonia research, and Table 2 lists the top 10 institutions depending on the number of publications. The most active institutions in the field of post-stroke pneumonia research were also overwhelmingly from the US, Germany, and England. The four institutions with the highest number of publications were Harvard University in the USA, Humboldt University of Berlin, Charité Universitätsmedizin Berlin, and Free University of Berlin in Germany, which also had the most citations and the highest H-index.

**Table 2:** The top 10 most active institutions that published articles.

**Rank**	**Institutions**	**Countries /Regions**	**Article counts**	**Total citations**	**Average citations per article**	**H-index**
1	Harvard University	USA	68	3,363	49.46	29
2	Humboldt university of Berlin	Germany	58	4,200	72.41	31
3	Charité universitätsmedizin Berlin	Germany	57	3,564	62.53	30
4	Free university of Berlin	Germany	57	3,564	62.53	30
5	University of London	England	40	1,890	47.25	20
6	University of Manchester	England	37	1,208	32.65	17
7	U.S. Department of veterans affairs	USA	37	2,056	55.57	20
8	Veterans health administration	USA	37	2,056	55.57	20
9	University of Toronto	Canada	34	2,577	75.79	17
10	Taipei medical university	Taiwan, China	32	489	15.28	12

**Figure 4:**
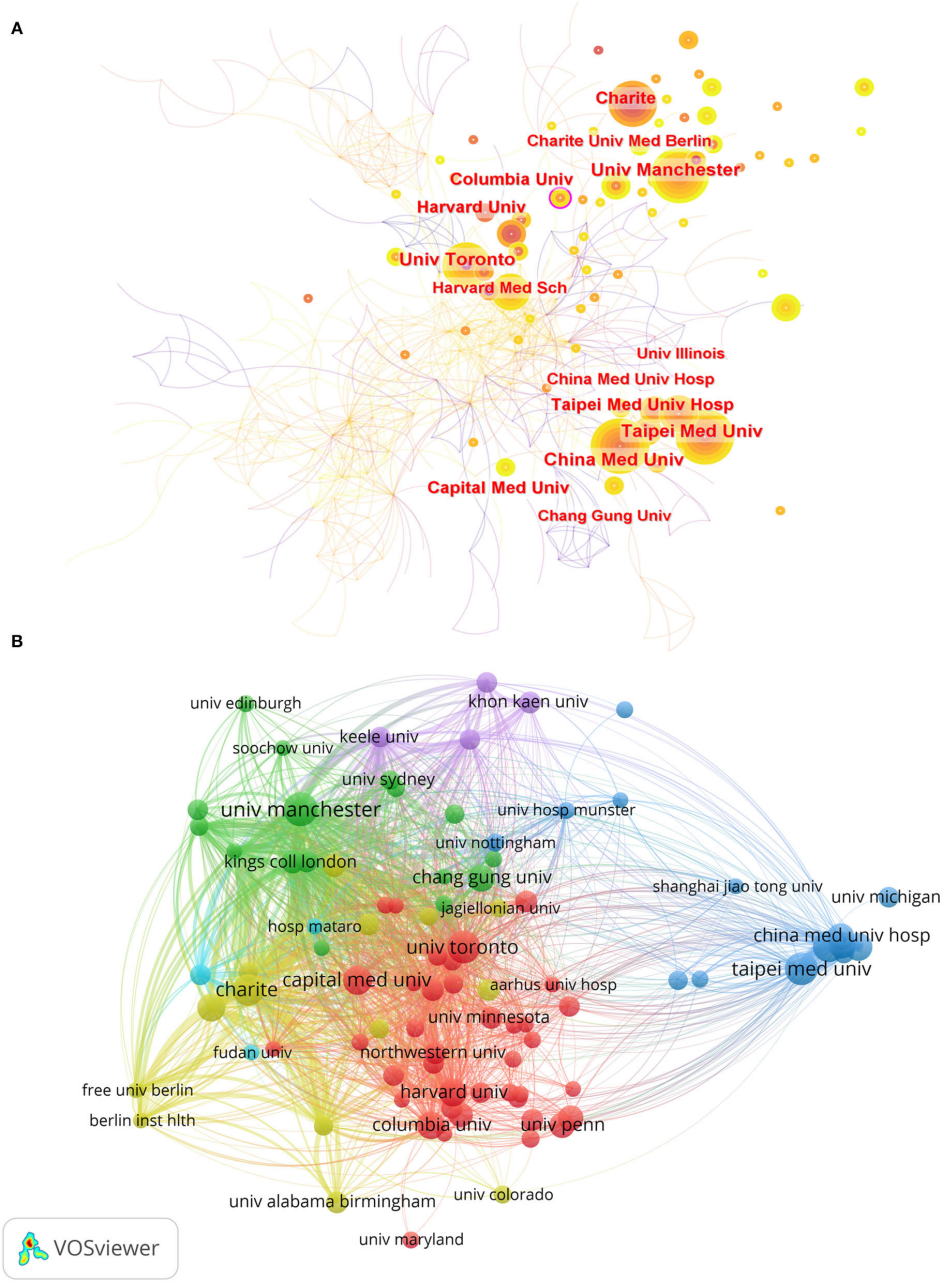
**(A)** The network cooperation map of institutions involved in post-stroke pneumonia research by using CiteSpace. **(B)** The institution's citation network visualization map created with VOS viewer.

Figure 4A shows the network cooperation map of institutions produced by CiteSpace. Each node in this figure represents a different institution. The larger the node indicates the more output of the institution, and the lighter the color of the node indicates the later the year the node is active. The highest centrality (0.13) was found at Columbia University, followed by the University of Manchester (0.09) and the University of Toronto (0.09). When the BC value ≥ 0.1, the node is a critical node and appears with a purple outer ring (31). From this, we can see that among all institutions, Columbia University plays the most important role in institutional cooperation, and this key node is shown in the figure with a purple outer ring. This paper also analyzes the citation of institutions with at least eight documents through the VOS viewer software. As can be seen in Figure 4B, there were 1,891 links and 90 nodes, which formed six different colored clusters. The following were the top three institutions having the highest TLS: Charité Universitätsmedizin Berlin (TLS = 935), University of Manchester (TLS = 935), and King's College London (TLS = 684).

### Analysis of funding agencies

**Figure 5:**
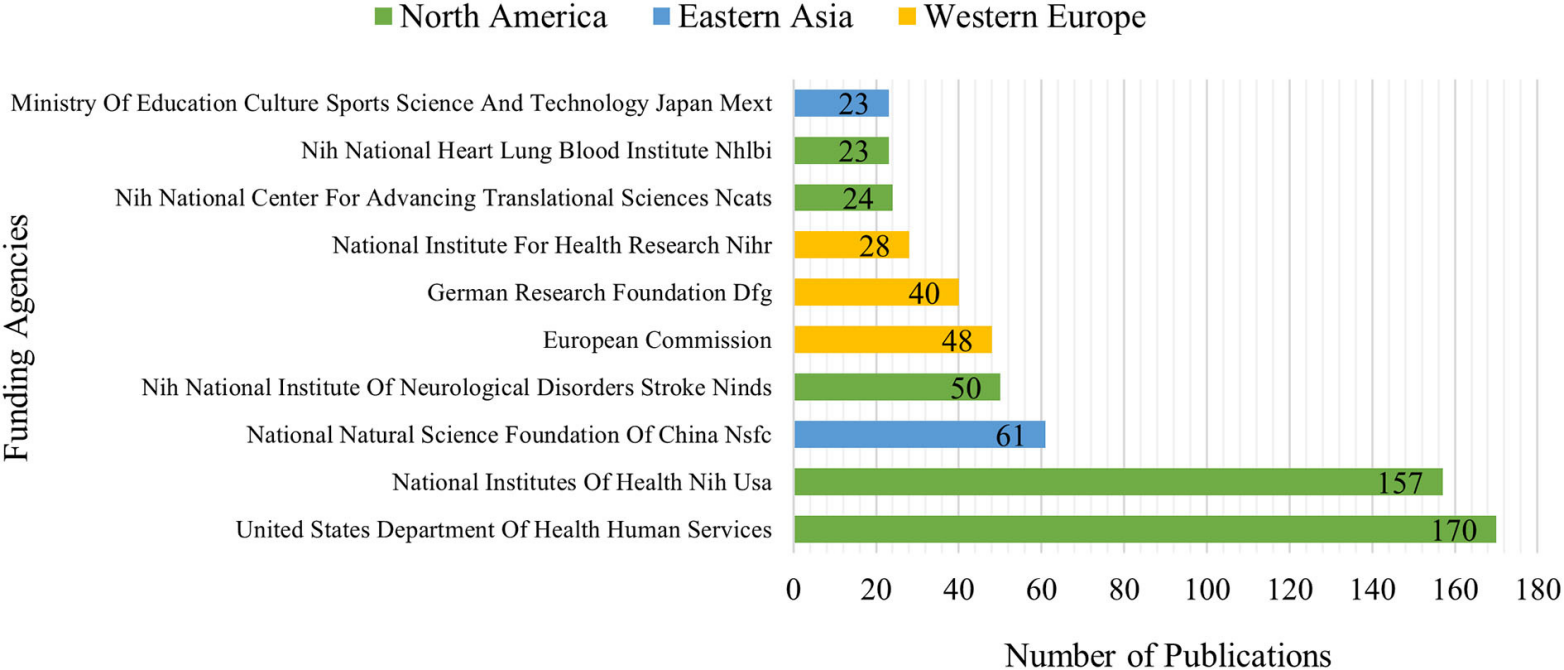
The top 10 funding agencies for the output in the post-stroke pneumonia field.

Figure 5 lists the top 10 funding agencies for the support of post-stroke pneumonia research. The United States Department of Health Human Services and the National Institutes of Health (NIH USA) were in first and second places, respectively. The top two funding agencies in the USA have supported over 100 studies on post-stroke pneumonia, far more than any other agency. Geographically, institutions in North America had funded the most publications, followed by Western Europe and East Asia.

### Analysis of authors

The top 10 productive authors had published 216 articles, and they accounted for 13.97% of the total number of publications (Table 3). A number of four of the top 10 productive authors were from Germany: Meisel A, Meisel C, Dziewas R, and Dirnagl U, in the first, second, seventh, and ninth positions, respectively. Also, three of the German authors (Meisel A, Meisel C, and Dirnagl U) were from Charité Universitätsmedizin Berlin. These authors (especially Meisel A and Meisel C, the most published authors), as top authors in the field of post-stroke pneumonia, have co-authored *Stroke-induced immunodeficiency promotes spontaneous bacterial infections and is mediated by sympathetic activation reversal by poststroke T helper cell type 1-like immunostimulation* (32) and *Central nervous system injury-induced immune deficiency syndrome* (33). These two articles, both of which were published earlier and have more than 600 citations, were the first to characterize stroke-induced immunodeficiency leading to severe bacterial infections (32) and provided a full explanation of central nervous system injury-induced immunodepression (CIDS) (33). This is a cornerstone for the study of the mechanisms of post-stroke pneumonia and has important implications for improving treatment (details of which will be mentioned in Cluster 3 below).

**Table 3:** The top 10 most productive authors in the field of post-stroke pneumonia.

**Rank**	**Author**	**Count**	**Countries /Regions**	**Institutions**	**H-index**	**Total citations**
1	Meisel A	41	Germany	Charite Universitatsmedizin Berlin	56	11,741
2	Meisel C	24	Germany	Charite Universitatsmedizin Berlin	47	9,755
3	Liao CC	23	Taiwan, China	Taipei Medical University	21	1,548
4	Chen TL	22	Taiwan, China	Taipei Medical University	34	3,579
4	Smith CJ	22	England	University of Manchester	41	5,453
6	Yeh CC	20	Taiwan, China	Taipei Medical University	4	48
7	Dziewas R	18	Germany	University of Munster	40	5,066
8	Montaner J	18	Spanish	University of Sevilla	75	20,179
9	Dirnagl U	14	Germany	Charite Universitatsmedizin Berlin	14	1,240
10	Wang YJ	14	China	Huazhong University of Science & Technology	45	7,244

**Figure 6:**
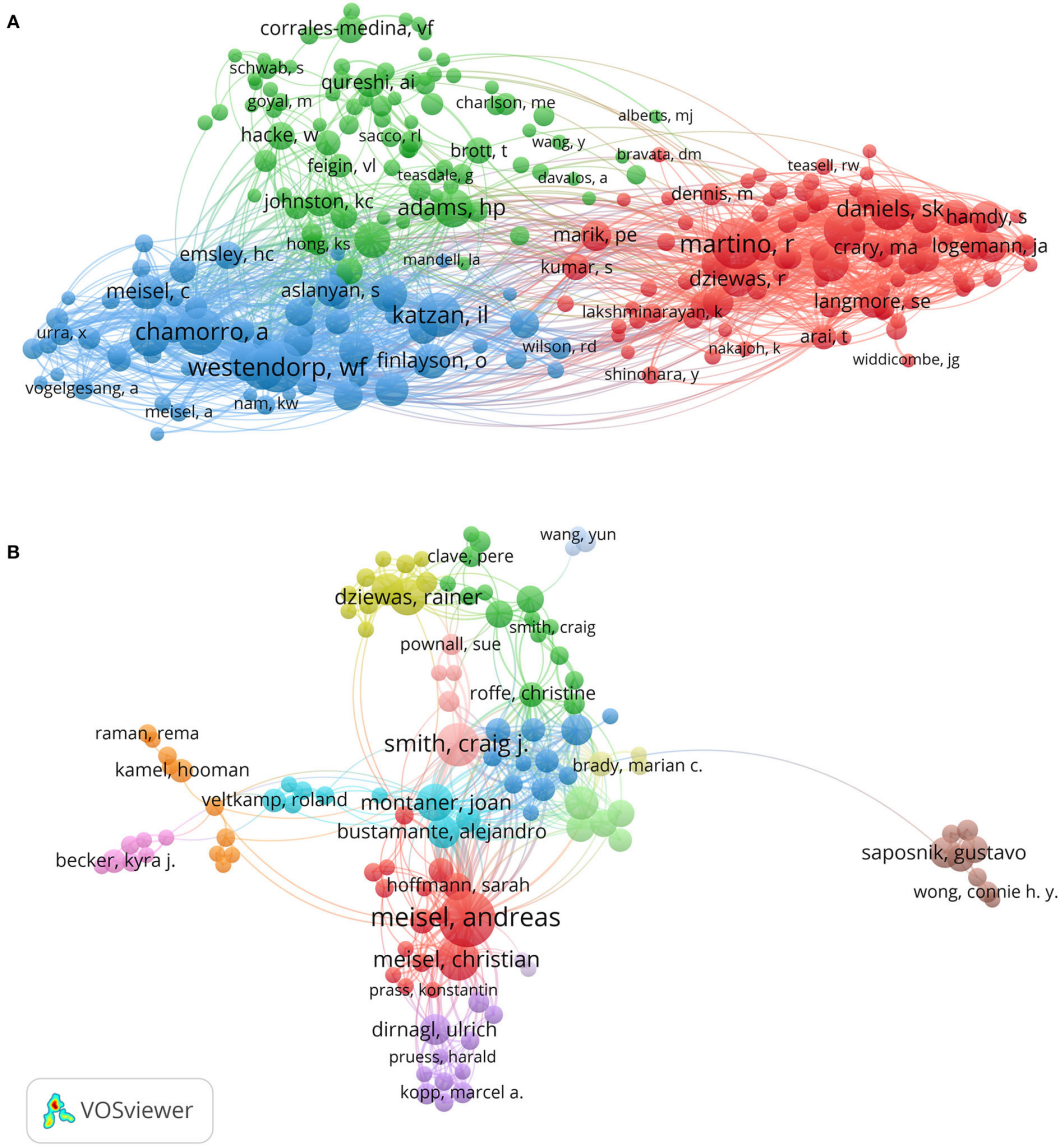
The co-citation analysis **(A)** and the co-authorship analysis **(B)** of the authors participated in post-stroke pneumonia research conducted by VOS viewer.

The co-citation network map of authors with at least 20 citations (Figure 6A) contains a total of 245 nodes present as authors, along with 16,445 links and 3 clusters. The top three authors with the highest TLS were Westendrop WF (TLS = 4,699), Martino R (TLS = 4,551), and Chamorro A (TLS = 4,375). Authors with at least three documents and at least 50 citations were visualized, resulting in 117 nodes that existed in the co-authorship network map of authors (Figure 6B). The nodes in this figure represented authors as in Figure 6A, but the difference is that the links between the nodes were dependent on the collaboration between the authors, rather than two authors whose articles were cited by others at the same time. The top three authors with the highest TLS were Meisel A (TLS = 178), Smith CJ (TLS = 95), and Montaner J (TLS = 85), who were at the center of the collaborative relationship. Overall, the distribution of nodes was scattered, suggesting that collaboration among authors in this field was not very close.

### Analysis of journals

In Table 4, the top 10 most productive journals are listed, most of which are from the USA. The journal with the highest number of publications was *JOURNAL OF STROKE CEREBROVASCULAR DISEASES*, followed by *STROKE*. Although *STROKE* had one less publication than the first place, it had the highest impact factor (IF). According to the Journal Citation Reports (JCR) 2020, the impact factor of the top 10 journals ranged from 1.889 in *MEDICINE* to 7.914 in *STROKE*.

**Table 4:** The top 10 most productive journals between 1998 and 2021.

**Rank**	**Journal title**	**Countries /Regions**	**Article Counts**	**Percentage** ** (*n*/1,546)**	**IF (2020)**	**Quartile in category**
1	Journal of stroke cerebrovascular diseases	USA	77	4.98%	2.136	Q4
2	Stroke	USA	76	4.92%	7.914	Q1
3	PLoS ONE	USA	44	2.85%	3.240	Q2
4	Dysphagia	USA	36	2.33%	3.438	Q1
5	Cerebrovascular diseases	USA	29	1.88%	2.762	Q3
6	Frontiers in neurology	Switzerland	26	1.68%	4.003	Q2
7	Neurocritical care	USA	26	1.68%	3.210	Q3
8	BMC neurology	England	25	1.62%	2.474	Q3
9	International journal of stroke	England	23	1.49%	5.266	Q1
10	Medicine	USA	23	1.49%	1.889	Q3

**Figure 7:**
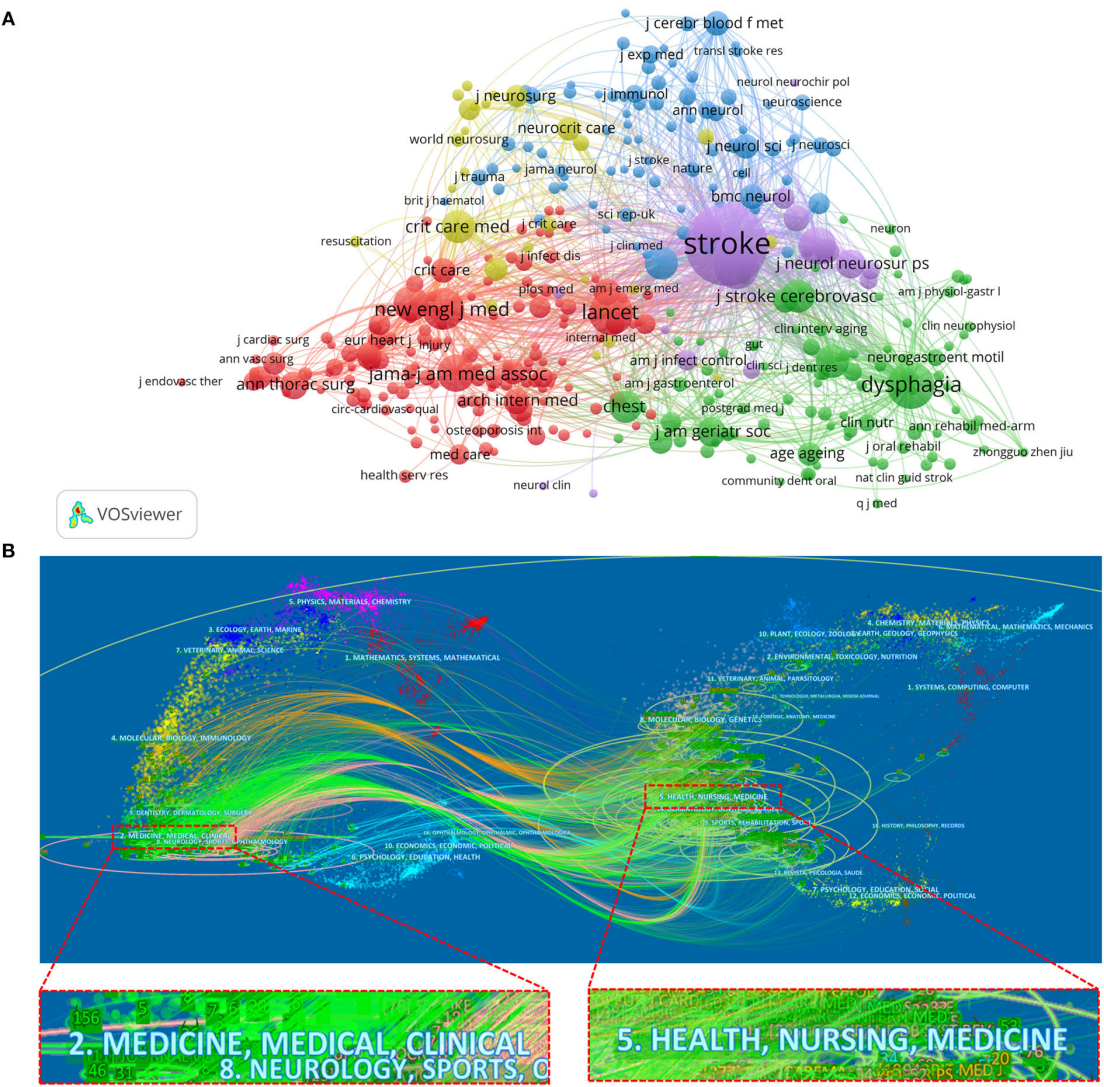
**(A)** The network visualization map of journals for co-citation analysis performed by VOS viewer. **(B)** The dual-map overlay of the journals involved in post-stroke pneumonia research generated by CiteSpace.

Figure 7A shows a network visualization map of the journal co-citation analysis, which visualized journals with at least 20 citations in the field. The top three largest TLS were *STROKE* (TLS = 172,131), *DYSPHAGIA* (TLS = 51,466), and *LANCET* (TLS = 45,361). The TSL of *STROKE*, as the first place, still far exceeded that of the other journals. The dual-map overlay of the journals shows the subject distribution of journals (Figure 7B). The left side of the map represents the citing journals; the right side of the map represents the cited journals; the colored lines indicate the citation relationship between articles in the citing journals and articles in the cited journals, and the whole figure can show the citation process in a complete way (34, 35). These subjects were surrounded by elliptical circles. The length of the vertical axis of the ellipse is determined by the number of papers published in the journal, and the length of the horizontal axis is determined by the number of authors. From the overall view of the map, the focus of published articles was on journals in the fields of medicine, medical, and clinical, whereas the cited journals were focused on a relatively broader range, with journals in the fields of health, nursing, and medicine being cited more often.

### Analysis of references

**Figure 8:**
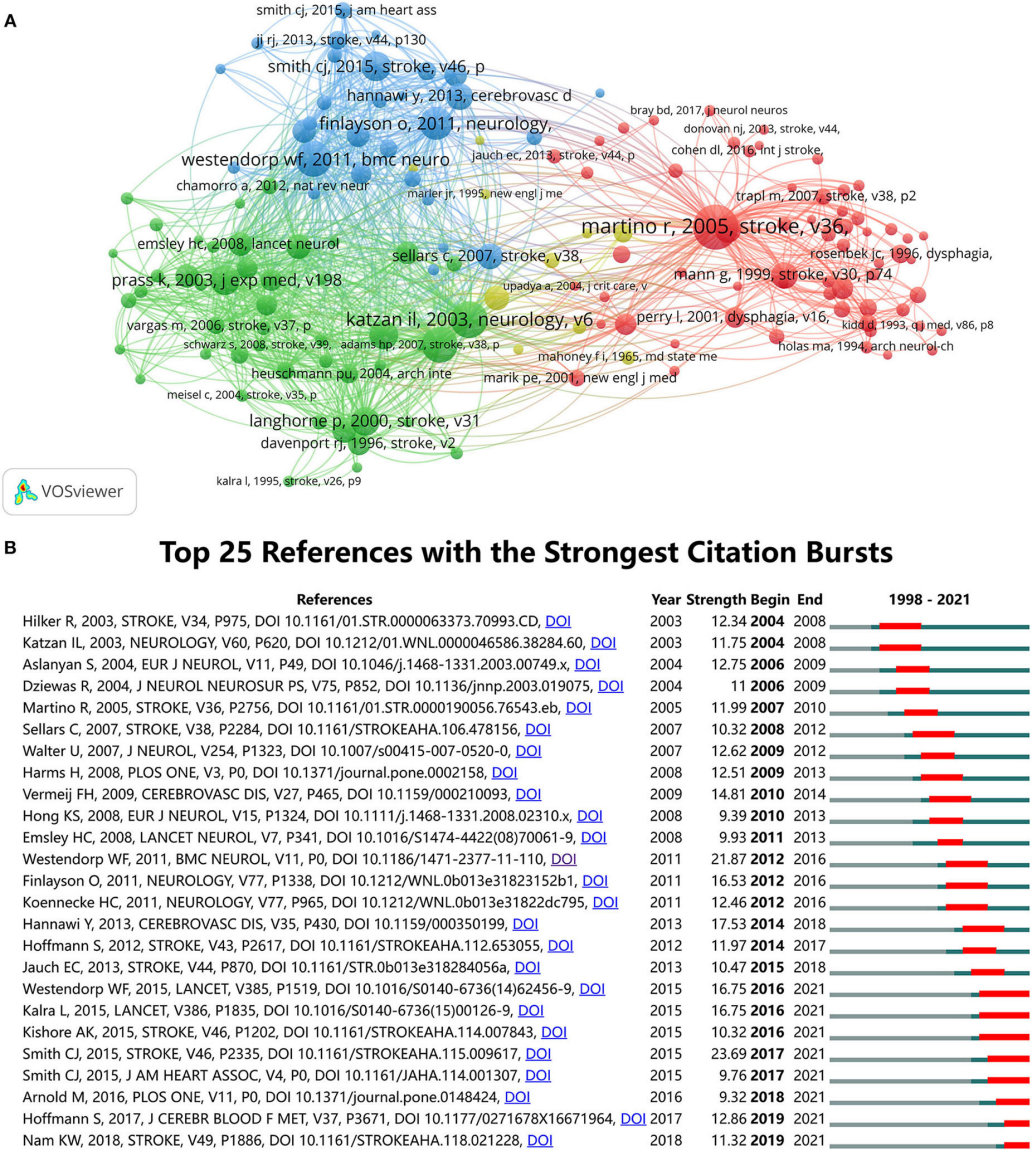
**(A)**. Document co-citation network visualization map generated by VOS viewer. **(B)** Top 25 references with the strongest citation bursts on post-stroke pneumonia research generated by CiteSpace.

Co-citation analysis of references is of great value to a field of academic research. In this paper, we used VOS viewer to generate the document co-citation network visualization map (Figure 8A) and listed the top 10 most cited articles related to post-stroke pneumonia in Table 5. There were 148 publications with at least 20 citations in the network, which were grouped into four clusters (colored red, blue, green, and yellow). Among the clusters, the total strength of the co-citation links with other cited references was calculated. The highest TSL was for an article published by Martino et al. (36) (TLS = 1,848) in 2005, followed by Katzan et al. (6) (TLS = 1,818) in 2003, Hilker et al. (2) (TLS = 1,688) in 2003, and Westendorp et al. (7) (TLS = 1,698) in 2011. All four of these papers also ranked among the top 10 most cited articles (Table 5). A more in-depth look at these articles also revealed that eight of the 10 articles were published between 2003 and 2006, and four were published in *STROKE*. The most cited article in the field of post-stroke pneumonia was a review published by Martino et al. (34) in *STROKE*. This article pointed out that dysphagia is one of the common symptoms of stroke. Through a systematic review of the published literature, it was accurately estimated that the presence of dysphagia significantly increases the risk of pneumonia in patients. Therefore, this article could be of value and help in research on the prevention of pneumonia through early interventions for dysphagia.

**Table 5:** The top 10 articles with the most citations in the field of post-stroke pneumonia.

**Rank**	**Title**	**Total citations**	**First author**	**Publication Year**	**Journal**
1	Dysphagia after stroke - Incidence, diagnosis, and pulmonary complications	997	Martino R	2005	Stroke
2	Stroke-induced immunodeficiency promotes spontaneous bacterial infections and is mediated by sympathetic activation reversal by poststroke T helper cell type 1-like immunostimulation	636	Prass K	2003	Journal of experimental medicine
3	Central nervous system injury-induced immune deficiency syndrome	599	Meisel C	2005	Nature reviews neuroscience
4	Impact of medical complications on outcome after subarachnoid hemorrhage	384	Wartenberg KE	2006	Critical care medicine
5	Post-stroke infection: A systematic review and meta-analysis	393	Westendorp WF	2011	BMC neurology
6	The effect of pneumonia on mortality among patients hospitalized for acute stroke	364	Katzan IL	2003	Neurology
7	Formal dysphagia screening Protocols prevent pneumonia	326	Hinchey JA	2005	Stroke
8	Medical and neurological complications of ischemic stroke - Experience from the RANTTAS trial	307	Johnston KC	1998	Stroke
9	Predictors of in-hospital mortality and attributable risks of death after ischemic stroke – The German Stroke Registers Study Group	287	Heuschmann PU	2004	Archives of internal medicine
10	Nosocomial pneumonia after acute stroke – Implications for neurological intensive care medicine	269	Hilker R	2003	Stroke

We can observe the top 25 references with the strongest citation bursts on post-stroke pneumonia research from Figure 8B. Of these, the two articles with the strongest burst were *Diagnosis of Stroke-Associated Pneumonia: Recommendations From the Pneumonia in Stroke Consensus Group* published by Smith et al. (3) in 2015 and *Post-stroke infection: A systematic review and meta-analysis* published by Westendorp et al. (7) in 2011. Both articles exceeded 20 in strength. In the first article, the Pneumonia in Stroke Consensus Group reached a consensus on terminology and diagnostic criteria for stroke-associated pneumonia (3), and the second article clarified the incidence of post-stroke pneumonia and its significant correlation with death (7), both of which had significant contributions to the study of post-stroke pneumonia and thus became the strongest citation bursts. From 1998 to 2021, the first burst occurred in 2004 due to an article published by Hilker et al. (2) and an article published by Katzan et al. (6) in 2003. The two most recent bursts occurred in 2019 and have continued to this day. This figure also shows that a total of eight articles are now still in the ongoing burst phase.

### Analysis of keywords

#### Analysis of temporal evolution in keywords

**Figure 9:**
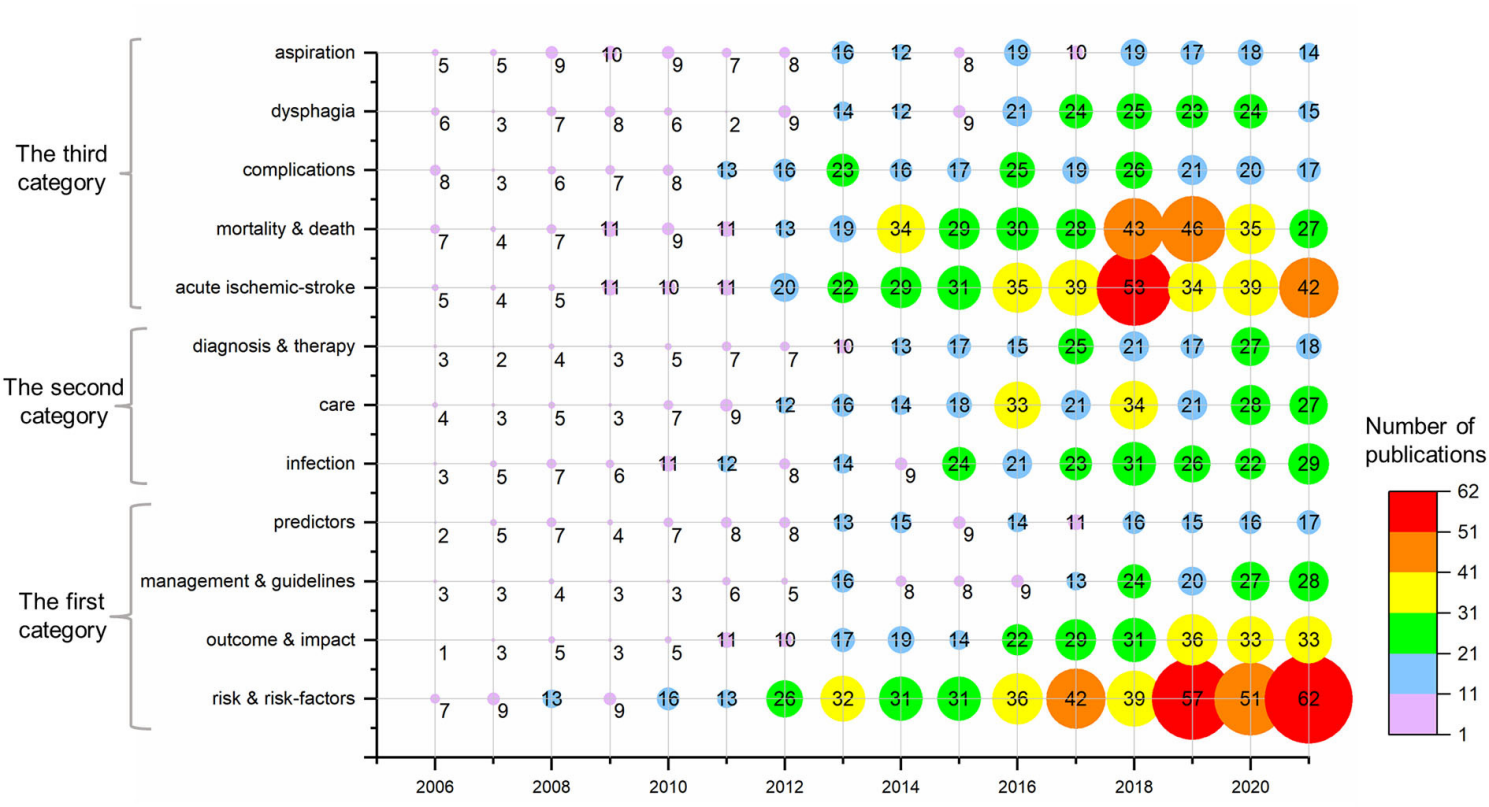
Bubble charts for 12 high-frequency topic keywords.

The evolution of keywords in time series can help to understand the development of the field at different times and predict future directions. A high-frequency topic keywords bubble chart from 2006 to 2021 is shown in Figure 9.

First, we sorted the keywords by the frequency of occurrence and then focused on the top 40 keywords with the highest frequency of occurrence. After that, we invited two neurologists to guide us. By combining some similar keywords and retaining the most representative topics in the field of post-stroke pneumonia as much as possible, we finally summarized the high-frequency keywords appearing in the field of post-stroke pneumonia into 12 hot topics. The numbers within the bubbles indicate that the number of times the hot topic has appeared in articles within a year. The size of the bubbles gives a clear indication of the evolution of these keywords over the years, and thus, the topics could be divided into three categories. The first category includes the following hot topic keywords: “risk & risk-factors,” “outcome & impact,” “management & guidelines,” and “predictors.” The number of articles related to the hot topics included in the first category has been increasing in the recent years, so these topics are the most worthy of our attention. According to the current situation shown in the chart, these topics will receive more attention within this field in the next few years. The second category includes “infection,” “care,” and “diagnosis & therapy.” They first kept increasing in frequency and then entered a plateau after 2015, with the annual frequency of appearance fluctuating slightly in the recent years. This suggests that they are still being paid attention to in the recent years. The third category includes “acute ischemic-stroke,” “mortality & death,” “complications,” “dysphagia,” and “aspiration.” Although they once had a high level of interest, they have seen a marked decline in popularity in the last 3 to 4 years.

#### Analysis of co-occurrence in keywords

**Figure 10:**
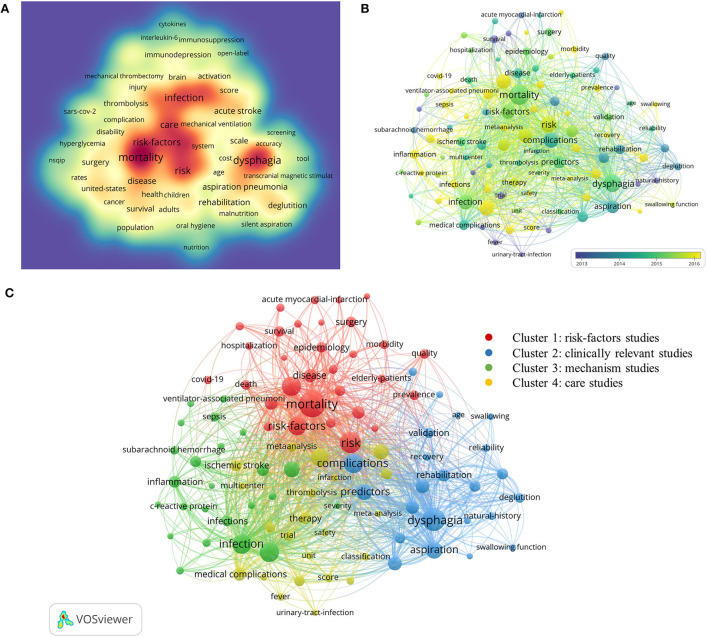
**(A)** The density visualization map of 240 keywords with more than 10 co-occurrences. **(B)** The overlay visualization map of 100 keywords with more than 20 co-occurrences. **(C)** The network visualization map of four clusters of 100 keywords created by VOS viewer.

In bibliometrics, analysis of frequently occurring keywords can disclose changing themes and trends, which are essential for understanding the developments in the field. In this paper, keywords were extracted from the titles and abstracts of all the documents searched, and the density visualization map (Figure 10A) was generated for keywords that co-occurred more than 10 times. The more times the keyword appears, the darker the color. A total of 240 keywords were present as nodes, with the exception of “stroke” and “pneumonia,” which were included in the search terms. The keywords with the highest number of co-occurrences were “mortality,” “dysphagia,” “risk,” “outcomes,” “complications,” and “infection.” Figure 10B shows the overlay map of the keywords, in which a total of 100 nodes represent the keywords that co-occurred more than 20 times. The VOS viewer software can use different colors to mark the keywords depending on the average appearing year (AAY) of keywords. Purple indicates keywords that appear relatively early in the limited time frame, followed by green, and the most recent occurrence is yellow. There has been a trend toward balanced growth in all four clusters during these years, and some new keywords have emerged in all of them. The high-frequency keywords (co-occurred more than 20 times) were presented as 100 nodes and 3,048 links in the network map (Figure 10C). Additionally, these keywords were grouped into four different colored clusters. In the Discussion section, we will elaborate on these four clusters.

## Discussion

### Research trends and knowledge structure of global publications

The chronological distribution of publications reflects, to some extent, the state of research, the level of research, and the pace of development in the field. Citations reflect the overall impact of a researcher's scientific output, with the number of citations per paper reflecting the average impact (37). Stroke is a serious cerebrovascular disease that has received widespread attention worldwide. As one of the major complications of stroke, post-stroke pneumonia has received increasing attention in this area due to its increased incidence of adverse events, mortality, and health care costs for patients (6, 38, 39). We can therefore assume that the number of publications on post-stroke pneumonia research will increase further, and this area is likely to enter a more prosperous phase in the near future.

From the perspective of countries/regions, the USA was far ahead of other countries in terms of both the number of publications and citations, so it can be said that it has been in a dominant position in this field. It is well-known that scientific research and innovation require significant financial, human, and material support. The United States Department of Health Human Services and the National Institutes of Health (NIH United States) were the funding agencies that funded the most research projects in this field, both from the United States and in far greater numbers than others. Therefore, support from funding agencies is one of the reasons why the United States has achieved high academic status in this field. At the same time, greater financial support could motivate high-level institutions to contribute to scientific research.

Based on a survey of author information, it could be found that most of these productive German authors also had the highest H-index. In total, three of them, Meisel A, Meisel C, and Dirnagl U, were associated with several highly productive German institutions in the analysis of the institution section above: Humboldt University of Berlin, Charité Universitätsmedizin Berlin, and Free University of Berlin. This suggests a complementary relationship between quality academic institutions and highly productive scholars. The three authors from Taipei Medical University also had a more significant number of publications, but the H-index and citations of these authors were low, indicating that the quality of their publications should be improved.

Highly cited articles are generally high-quality research in a field that has had a large impact on innovation and discovery. These publications are also considered essential reading material for academics preparing to work on the subject. The analysis of references shows that most high-quality articles were published in *STROKE*, which has dominated the field in terms of quantity and quality. As the top co-citation journal in the field, *STROKE* has a significant impact on academics studying post-stroke pneumonia, and its publications can also be used as the authoritative references (40).

The temporal evolution of the keywords shows that the topics “risk & risk-factors,” “outcome & impact,” “management & guidelines,” and “predictors” are gaining increasing attention in the field of post-stroke pneumonia. Therefore, academic research in these directions should be further expanded in the field of post-stroke pneumonia in the future. The trend in hot keywords and topics over time shows that research hotspots have undergone a transition from early studies on the mechanisms and clinical manifestations of post-stroke pneumonia to later studies on clinical treatment and prevention.

### Research hotpots of global publications

In bibliometrics, the study of frequently occurring keywords might reveal shifting patterns and major themes that are essential for understanding developments in the field. Combining Figures 9, 10C, it can be seen that current high-frequency keywords in the field of post-stroke pneumonia were grouped into four clusters. After aggregating and analyzing all the keywords involved in the four clusters, we again imported the data into CiteSpace and performed keyword clustering analysis, which resulted in silhouette S = 0.7307 and modularity Q = 0.3519. It is generally considered that silhouette S > 0.7 means that the clustering is convincing and modularity Q > 0.3 means that the clustering structure is significant (41, 42). We confirmed that the four clusters here are reasonable. Then, we arrived at the final four cluster designations through the guidance of three neurologists and the unanimous approval of all the authors of this paper after reviewing the relevant literature and discussing them, which are: cluster 1 (risk-factors studies of post-stroke pneumonia) is the largest cluster, containing a total of 31 keywords, mainly related to the terms “risk-factors,” “mortality,” “outcomes,” etc.; cluster 2 (clinically relevant studies of post-stroke pneumonia) contains 24 keywords, the main ones being “dysphagia,” “aspiration,” “predictors”; cluster 3 (mechanism studies of post-stroke pneumonia) contains 23 keywords, including the following: “infection,” “inflammation,” “acute ischemic-stroke,” etc.; cluster 4 (care studies of post-stroke pneumonia) contains 22 keywords, with emphasis on “care,” “meta-analysis,” and “prevention.”

#### Cluster 1: Risk-factor studies of post-stroke pneumonia

This cluster is the largest of the four clusters. As research in the field of post-stroke pneumonia has gained interest, some risk-factors have been demonstrated to be strongly associated with the incidence and adverse outcome of post-stroke pneumonia. Analyzing the results of previous studies, two categories of risk-factors can be identified for post-stroke pneumonia: self-inflicted factors and medical-induced factors. Self-inflicted factors include age, sex, stroke site, stroke type, stroke severity, previous medical history (diabetes, coronary artery disease, hypertension), smoking history, impaired consciousness level, dysphagia, chronic alcohol consumption, atrial fibrillation, and hypoproteinemia (43–50); medical-induced factors are mainly caused by clinical medication and medical practices, such as the use of mechanical ventilation, nasogastric tube, prophylactic antibiotics, proton pump inhibitor (PPI), and H2 receptor antagonists (47, 51, 52). These risk factors may lead to a weakened cough reflex, imbalance in the bacterial flora of the oral pharynx, colonization by pathogenic bacteria, regurgitation, aspiration, and suppression of the immune response, all of which increase the risk of post-stroke pneumonia and improve the mortality of patients. Although there is a large body of research on the risk factors for post-stroke pneumonia, the factors involved vary from study to study and the results vary, so it is not possible to standardize the risk factors for post-stroke pneumonia at this time.

Several risk assessment scales for post-stroke pneumonia have been proposed in the recent years, such as the A2DS2 score, ISAN score, AIS-APS score, and PANTHERIS score, which have been shown to be accurate in predicting the risk of post-stroke pneumonia (53–56). It may help in the early clinical selection of interventions to control the risk of post-stroke pneumonia.

By exploring the risk factors for post-stroke pneumonia, we can help clinicians raise awareness of the occurrence of post-stroke pneumonia and predict the causative factors more accurately, so that prevention and treatment can be effectively selected on time. This has important implications for improving the prognosis of patients with post-stroke pneumonia, shortening the length of hospital stay, and reducing the financial burden on patients.

#### Cluster 2: Clinically relevant studies of post-stroke pneumonia

Clinical symptoms of dysphagia could contribute to the development of post-stroke pneumonia (57). Data show that up to 50% of patients with post-stroke presented clinically with dysphagia, and nearly half of them were at risk of aspiration (58). Dysphagia after stroke is mainly associated with oropharyngeal dysfunction, with dysphagia being characterized by sluggish rather than diminished hyolaryngeal motions during swallowing (59). Dysphagia is a significant risk factor for pneumonia on the 1 day after stroke, increasing the risk of pneumonia in patients with stroke more than 3-fold; when dysphagia leads to a diagnosis of aspiration, patients are 11 times more likely to develop pneumonia (36), which means that aspiration in patients with stroke with dysphagia significantly increases the incidence of pneumonia. Exploration in this area has long been one of the focuses of post-stroke pneumonia. Swallowing dysfunction increases the risk of post-stroke pneumonia by the following mechanisms: (1) Patients with swallowing dysfunction have reduced swallowing and cough reflexes, and foreign bodies from the oral and nasal cavities can easily enter the trachea and lower airways by mistake. (2) Patients with dysphagia require nasal feeding with a nasal feeding tube, which can irritate the trachea and stomach, triggering adverse reactions such as nausea and vomiting, which in turn increase the risk of aspiration.

Since the clinical presentation of dysphagia increases the incidence of post-stroke pneumonia, further research on this topic may be helpful in the prevention of post-stroke pneumonia. This has led to several studies in this area, which have found that early dysphagia screening (EDS) is effective in reducing the incidence of post-stroke pneumonia (60, 61), and that early swallowing training is effective in reducing pulmonary complications (62). This will likely be important in specifying standardized preventive measures and management strategies for such patients in the clinical setting. However, some other studies have questioned this, arguing that there is insufficient data to demonstrate the impact of dysphagia screening on reducing post-stroke pneumonia and mortality (49, 60, 63). Overall, the study of dysphagia has attracted more attention in the field, but there is still some rooms for research in clinical practice. More trials will be needed in the future to compare the clinical effectiveness and feasibility of different dysphagia screening methods for the prevention of post-stroke pneumonia, so that preventive measures can be better adapted.

#### Cluster 3: Mechanism studies of post-stroke pneumonia

Immunosuppression is an important intrinsic mechanism for inducing post-stroke pneumonia, and research on this aspect is now well established. The systemic immune response after stroke protects brain tissue by avoiding further inflammatory stimuli, but it can cause immunosuppression, resulting in stroke-induced immunosuppression syndrome and infection. In 2003, Prass et al. discovered through animal experiments that an increase in local proinflammatory cytokines in cerebral ischemia leads to rapid and persistent suppression of cellular immune function (monocyte deactivation, lymphopenia, Th1/Th2 shift) by the activation of the sympathetic nervous system (SNS), thus reducing the body's immune function and predisposing it to infection (32). In 2005, Meisel et al. found that central nervous system (CNS) injury causes a disturbance in the normally balanced interaction between the immune system and the central nervous system, resulting in secondary immunosuppression and termed it CNS injury-induced immunodepression (CIDS) (33). Several research papers have shown that immune dysfunction can be triggered by stroke, suggesting that this is one of the mechanisms leading to the development of post-stroke pneumonia. Immune dysfunction following stroke may be associated with over-activation of the sympathetic and parasympathetic nervous systems and the hypothalamic–pituitary–adrenal (HPA) axis.

Based on the above, it has been suggested that by preventing the mobilization of T and B cells, which carry a cytokine death warrant to the brain, the ability to defend against systemic infectious injury could be restored, thereby reducing the probability of pneumonia. This requires the development or effective use of drugs that block early neural splenic activation and modulate brain antigen-specific immune cells (64). There is still some controversies about the methods and means of preventing and treating post-stroke pneumonia from an immunological perspective. Prevention of infection is a cornerstone of treating patients with stroke to improve long-term outcomes (65), and in the future, more research may be devoted to the therapeutic option of reversing post-stroke immune damage by boosting host immunity. There is still a lack of large, reliable studies on the use of immunomodulatory approaches to stroke prevention and treatment, and more clinical and animal studies are needed.

#### Cluster 4: Care studies of post-stroke pneumonia

Precise care for patients with stroke could be effective in preventing and treating post-stroke pneumonia. Current research on the care of post-stroke pneumonia has focused on feeding management, oral care, and drug management.

##### Feeding management

For patients with stroke with normal gastrointestinal function, the option of enteral nutrition can ensure nutritional intake and prevent malnutrition. For patients who cannot swallow, other enteral nutrition options such as nasogastric (NG) tube, oroesophageal (OE) tube, and percutaneous endoscopic gastrostomy (PEG) can be chosen early (66, 67). Of these, the NG tube is the most widely used, whereas the PEG is less commonly used clinically because it is an invasive treatment. Both the risks and benefits of these tube feeding modalities are present. Tube feeding has been recommended as an alternative technique of giving nutritional assistance, but it has been shown to increase the incidence of pneumonia and mortality after a stroke, rather than prevent it (68, 69). To avoid complications while feeding by tube in clinical practice, careful control of the amount, interval, position, and feeding method is required. Overall, enteral nutrition modalities, particularly the NG tube, have been tested and explored by some authors over the years. To prevent the development or aggravation of pneumonia, nursing staff should choose and use a relatively low-risk tube feeding method that takes into account the patient's particular circumstances. Of course, further studies with improved design are needed in this area, as well as more experimental data to compare the effectiveness and risk of various tube feeding procedures to find the best way to prevent post-stroke pneumonia.

##### Oral care

Oral care is one of the most effective ways to prevent and treat post-stroke pneumonia. It is reasonable to maintain good oral hygiene and dental plaque control in the early post-stroke phase. Specifically, in patients with stroke with dysphagia and impaired consciousness, stagnation of saliva and food in the mouth may occur (70) and lower tongue pressure and altered lateral movements may contribute to an increased risk of aspiration (71), thus increasing the incidence of post-stroke pneumonia. It is also worth noting that poor oral health can induce high levels of bacteria in the saliva, which can result in the development of pneumonia when inhaled due to dysphagia or other illnesses (72). As a result, by removing foreign bodies from the mouth, killing oral bacteria, and enhancing the oral environment, the risk of aspiration in patients with stroke can be lowered, thereby reducing the incidence of post-stroke pneumonia. Practice guidelines and clinical practice recommendations for oral care are continually being updated, and future research in the field of post-stroke pneumonia will provide more detailed instructions on oral hygiene and treatment for the care of post-stroke patients. In the future, more in-depth education tailored for nursing personnel will help to provide optimal care and ensure the implementation of evidence-based practice.

##### Drug management

Pneumonia is a common condition in post-stroke infections, and antibiotic therapy is one of the main treatments for infection and inflammation. However, antibiotic prescribing practices vary widely among healthcare systems (73), and there are few standardized antibiotic therapy guidelines for post-stroke pneumonia. It can be said that empirical antibiotic therapy is the basis of early treatment for post-stroke pneumonia. The pneumonia in stroke consensus (PISCES) group participants had agreed that there was insufficient evidence to recommend any specific antibiotic drug or class of antibiotic for the treatment of SAP (74). A standardized approach to the use of antibiotics may therefore be a key component of antibiotic management and clinical outcomes improvement. This area also needs to be actively pursued with more pathogenic testing to optimize anti-infective regimens and establish a unified standard.

The topic of whether prophylactic antibiotics can help to prevent post-stroke pneumonia is still up for debate. Available trials have concluded that the use of prophylactic antibiotics does not improve functional outcomes after stroke (51, 75) and may even be risky. Therefore, most of the findings so far do not recommend the prophylactic use of antibiotics clinically.

## Limitations

There are several drawbacks to this study that should be mentioned as well. The first is the literature retrieval form. Although the search terms were set up as comprehensively as possible, there were some areas of research that may not have been covered. Second, despite the use of a rigorous and well-structured research process, only publications published in the Web of Science database were chosen as data for analysis. Google Scholar and Scopus were not searched, which could have resulted in the omission of relevant papers. Furthermore, while objective results can be obtained in relevant research areas through bibliometric analysis, some of the underlying reasons for these results need to be further explored. The discussion and analysis presented in this article are intended to assist the reader in developing a clearer framework and implementing more effective strategies to gain a better understanding of post-stroke pneumonia. This article may also assist the research community in identifying existing research gaps, thus contributing to the discipline's direction in this area.

## Conclusion

In summary, we have seen a growing interest in the field of post-stroke pneumonia in the recent years, and it is predicted that more papers will be published in the coming years. The results of this study show that the main contribution came from the USA. At the same time, German professionals and research institutes have conducted in-depth research on this subject, whereas Asian countries such as China have contributed a substantial quantity of output. *STROKE*, with the high number of publications, IF, and TLS, was the most important journal in the field of post-stroke pneumonia. In addition, a co-occurrence analysis of keywords revealed that high-frequency keywords were grouped into four clusters: cluster 1 (risk factor studies of post-stroke pneumonia), cluster 2 (clinically relevant studies of post-stroke pneumonia), cluster 3 (mechanism studies of post-stroke pneumonia), and cluster 4 (care studies of post-stroke pneumonia). Combined with analysis of temporal evolution in keywords, it is revealed that the research hotspots in the field of post-stroke pneumonia have undergone a shift from early research on the mechanisms of post-stroke pneumonia to later research on clinical treatment and prevention.

## Data availability statement

The article/Supplementary material contains the original contributions given in the study; further questions can be sent to the corresponding author.

## Author contributions

XL proposed the topic and designed the overall article. JY processed and analyzed the data. CS summarized the data. All authors contributed to the article and approved the submitted version.

## Funding

This work was supported by the Tianjin Philosophy and Social Science Planning Project (grant number TJGL21-019) and the Natural Science Foundation of Tianjin (grant number 20JCZDJC00540).

## Conflict of interest

The authors declare that the research was conducted in the absence of any commercial or financial relationships that could be construed as a potential conflict of interest.

## Publisher's note

All claims expressed in this article are solely those of the authors and do not necessarily represent those of their affiliated organizations, or those of the publisher, the editors and the reviewers. Any product that may be evaluated in this article, or claim that may be made by its manufacturer, is not guaranteed or endorsed by the publisher.
